# Basal ganglia germinoma presenting with visual loss in male adult: A case report

**DOI:** 10.1016/j.ijscr.2024.110225

**Published:** 2024-08-29

**Authors:** Vega Pangaribuan, Tedy Apriawan, Muhammad Azzam

**Affiliations:** Department of Neurosurgery, Universitas Airlangga – Dr. Soetomo General Academic Hospital, Surabaya, East Java, Indonesia

**Keywords:** Basal ganglia tumor, Germ cell tumor, Craniotomy, Transcortical approach, Case report

## Abstract

**Introduction:**

Basal ganglia germ cell tumor (GCT) in an adult has been rarely reported Intracranial germ cells tumor usually occurs in the midline axis, involving pituitary, sellar region, or both. Only in rare circumstances GCTs developed in basal ganglia.

**Case presentation:**

A 27-year-old male came to our academic general hospital outpatient clinic with main complaint of progressive visual loss of his right eye which started a year ago. Despite his visits to the opthalmologist, the complaint worsen. A brain MRI revealed a large left basal ganglia tumor with involvement of the hypothalamus and uncus, causing pressure on the optic and occulomotor nerve. Interestingly, the patient had no decrease in motor function. Complaint of severe headache, persistent vomiting, and decrease of vision of his left eye prompt us to conduct an urgent craniotomy tumor excision. As the patient had no motor deficit prior to surgery, we chose to do a transcortical approach through the left kocher point, entering the left ventricle, and accessing the tumor from the floor of the frontal horn.

**Discussion:**

GCT is a rare and diverse group of tumors based on histology. It is more common in men and mostly affects the pediatric and adolescent populations. Usually located in the sellar or pineal regions, this tumor may infrequently spread to the basal ganglia. The gold standard for diagnosing germ cell tumors is histopathological analysis, particularly in regions of the basal ganglia with a wide range of potential aetiologies. Obtaining a biopsy sample surgically is difficult, particularly for patients whose motor function is preserved. The surgical strategy should be tailored to the patient's radiological and clinical results, ideally taking the surgeon's preferences into account as well.

**Conclusion:**

Basal ganglia germinoma in adult is a rare occurrence, and due to its location, the surgical approach to access the mass should be individualized in each patient. Transcortical approach from the left kocher's point was a safe and accessible approach for our patient.

## Highlights

• Basal ganglia germinomas commonly occurs in pediatric population.
• There is no prior report of this tumours developing in an adult whose main complaint is loss of vision.
• Accessing the basal ganglia tumor surgically is a challenge, especially in patient with intact motor function.
• Transcortical approach from the left kocher’s point was a safe and accessible approach for our patient.

## 1 Introduction

Germ cell tumor (GCT) is a rare intracranial tumor and histologically heterogenous. This tumors usually located in the midline, involving the suprasella (23 %–35 %) and the pituitary gland (37 %–66 %). On rare cases it may involve the basal ganglia region (0 %–8 %) [1].

The incidence of GCTs varies across the continents, with 0.5 / million in Israel, 0.6 million in United States, and 2.7 / million in japan [1]. Basal ganglia GCT is considerably more uncommon, and mostly occurs in children and teenagers. A study by Zhang et al., reporting that the median age of basal ganglia germ cell tumor is 12.5 years old, predominantly in males [2]. As basal ganglia responsible for controlling the motoric function, and also the control of behaviour, cognition and emotions the patients usually presented with symptoms of motor weakness and cognitive decline [3].

Diagnosing the origin of basal ganglia tumor can be quite of a challenge since it has diverse histopathological types and varying treatment outcomes. Other differential diagnosis includes astrocytomas, gangliogliomas, oligodendrogliomas, dysembryoplastic neuroepithelial tumor, anaplastic ependymomas, primitive neuroectodermal tumor, and lymphomas. As germ cell tumors incidence is higher in pediatric and young adults, Zhang et al. recommend routine examination of β-human chorionic gonadotropin (β-hCG) and A-fetoprotein (AFP) in these subgroups. The backbone treatment for GCTs are radiotherapy and chemotherapy [4]. Surgical management usually reserves to get tumor biopsy and tissue sampling, cerebrospinal fluid diversion in hydrocephalus, and those who suffer from acute visual deterioration and signs of elevated ICP [5].

## 2 Case presentation

**Fig. 1:**
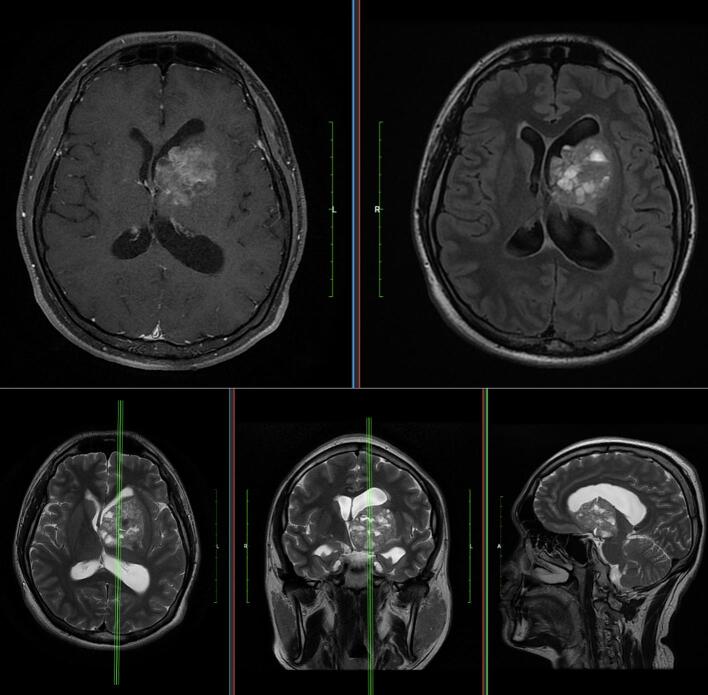
T1, T2, and T2 FLAIR weighted MRI revealing a mixed intensity tumors with multiple haemorrhages in the left basal ganglia region, sizing 4.1 cm × 4.0 cm × 4.2 cm. The mass causing a midline deviation of 10 mm to the contralateral side.

A 27-year-old male came to neurosurgical outpatient clinic of our academic general hospital with complaint of progressive visual loss from his right eye from one year prior. He had been going to the ophthalmologist but there is no improvement for his visual deterioration. Clinical examination revealed a Glasgow coma scale (GCS) of 15, anisocoric pupil, dilated right pupil with no light reflex from his right eye. His visual examination resulted in no light perception from his right eye and visual acuity of 6/6 for his left eye. There was no lateralization with motor score 5 through all 4 extremities. His brain MRI revealed a contrast enhancing mass in the left basal ganglia with involvement of the hypothalamus and temporal uncus, causing pressure on the optic and oculomotor nerve. (Fig. 1).

**Fig. 2:**
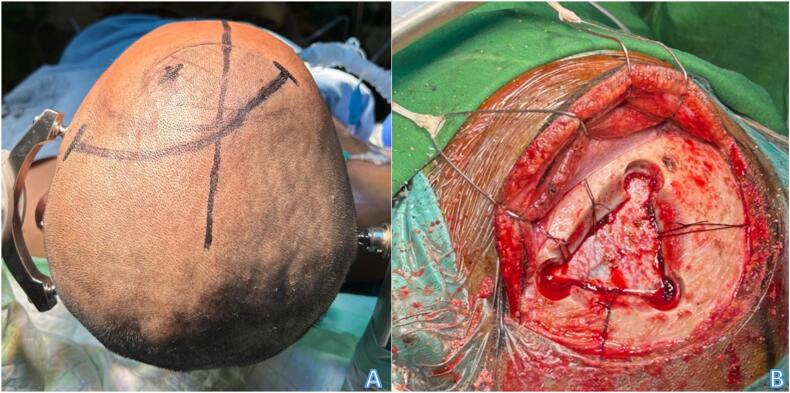
Curvilinear incision was made to access the left kocher's point (A). Exposure of the duramater following the bone removal (B).

Interestingly, the patient had no decrease in motor function. The patient came to the emergency department one week after the outpatient visit, with complaint of severe headache, persistent vomiting, and complaint of blurring of vision of his left eye. This prompt us to conduct an urgent craniotomy tumor excision. As the patient had no motor deficit prior to surgery, we chose to do a transcortical approach through the left kocher point, entering the left ventricle, and accessing the tumor from the floor of the frontal horn (Fig. 2).

**Fig. 3:**
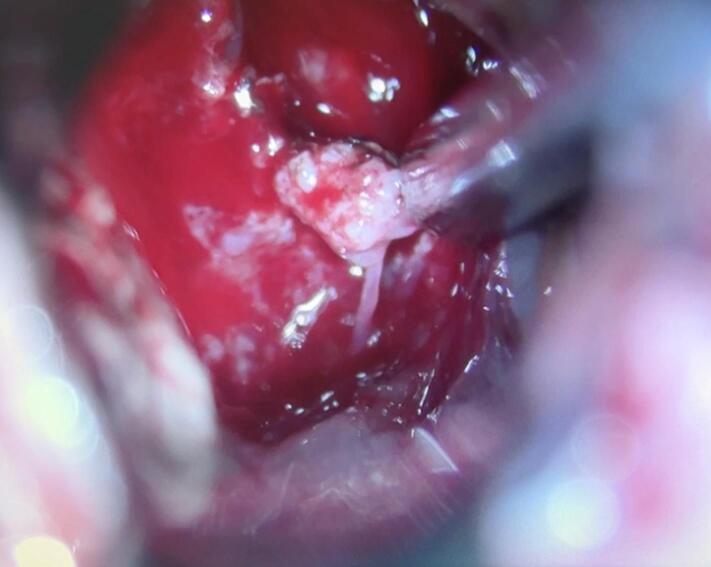
Intraoperative findings of the tumor mass. The tumor appears soft, greyish, and hypervascular.

The tumor was immediately identified. The mass was well differentiated, hypervascular, and fragile (Fig. 3). The aim of the surgery is to reduce the tumor mass and taking out a sufficient sample biopsy. The tumor was debulked using CUSA to minimize the manipulation to the surrounding normal brain tissue. We purposefully did a subtotal resection, to avoid damaging the intact basal ganglia function.

Following the surgery the patient reported his vision remains the same with no complaint of motor weakness. However 2 days following the surgery patient had steep increase of urine production. He was then treated with oral desmopressin acetate, and the polyuria complaint was controlled.

**Fig. 4:**
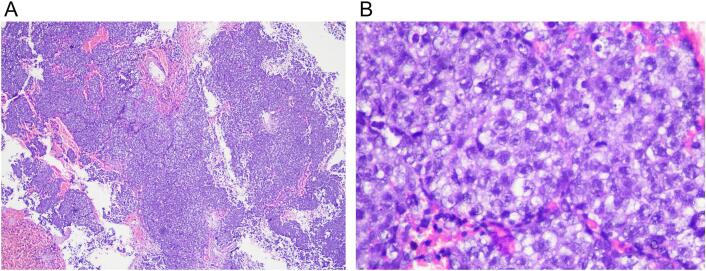
A pathological anatomy result with hematoxylin eosin staining, revealing tumor tissue arranged in sheets, some of the lobules are separated by thin fibrous connective tissue with infiltration of inflammatory cells of lymphocytes. There are proliferation of anaplastic cells, polygonal shaped, with round nuclei, rough chromatin, prominent nuclei, sufficient, eosinophilic, mitosis 17 out of 10 high-power field. Areas of necrosis and bleeding are visible.

The pathology anatomy result was germinoma (Fig. 4). His serum β-hCG was slightly elevated with normal level of AFP, which supported the diagnosis of germinoma. The patient was immediately referred for radiotheraphy, as germinoma is a radiosensitive tumor.

**Fig. 5:**
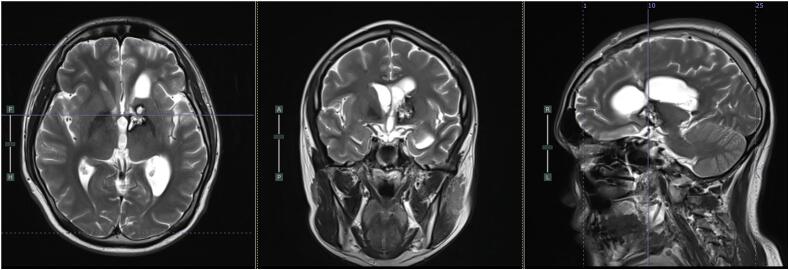
T2 weighted MRI showed iso to hyperintense mass in the basal ganglia sizing 2.2 × 1.2 × 2.2 cm. The mass appears smaller compared to the previous imaging.

The patient had been followed up for 6 months following the surgery. He had finished his radiotherapy course of 2 Gy each fraction for 15 times and additional booster 2 Gy each fraction for 10 times. His visual function remains the same, with no complaint of motor function. The MRI Evaluation after the radiotherapy showed that the tumor mass appears smaller (Fig. 5). He was planned for follow-up MRI and is willing to comply with regular outpatient visits.

The patient and his family feels that the doctor's recommendations were all for the patient's best interests and is always grateful for them during treatment. In order to treat the patient's condition, the patient's family trusts the doctor to do whatever is necessary. The patient's family expresses their appreciation for the doctor's care as well as the fact that the patient's condition is gradually getting better.

This case report has been reported with Surgical Case Report Guidelines (SCARE) 2023 [6].

## 3 Discussion

GCT is a rare and histologically heterogenous group of tumors. It is predominantly involves pediatric and adolescent population, and had a higher prevalence in male [7]. Peak age of diagnosis of this tumor is between 10 and 12 years old and 90 % of all cases were diagnosed before 20-years old [8]. This tumor location usually found in sellar or pineal region, and rarely it may involve the basal ganglia region [1].

GCTs tumor were divided into two main groups: the germinomas and non germinomatous germ cell tumors (NGGCT). The NGGCTs are histologically differentiated into embryonal cell carcinoma, yolk sac tumor, choriocarcinoma and mature or immature teratoma. Germinomas is the most common subtype, comprising around two thirds of all GCTs. It is derived from totipotent germ cells and had better prognosis than the other subtypes, since most of germinomas were radiosensitive. Mature teratomas can be managed with complete resection. However, other NGGCTs are associated with worst prognosis with 5-year survival rates at 9–49 % even with chemotherapy and radiotherapy [9].

A study by Wei Fu et al., suggest a routine tumor markers examination such as B-HCG and AFP for basal ganglia tumor. Tumor biopsy is reserved only in those who had no elevation of the tumor markers or non-secreting germ cell tumor [4].

Histopathological diagnosis for the basal ganglia germ cell tumors remains as the gold standard, especially in basal ganglia regions which has vast possible etiopathologies. Therefore, surgical approach to retrieve biopsy sample becomes a challenge, especially in patients with preservation of motor function. The surgical approach should be individualized based on radiological and clinical findings of the patient, preferably also considering the surgeon's preference. A study by Yang et al., reported the use of right trans-frontal lateral ventricle approach, transsylvian transinsular approach [10]. Kumar et al. reported the use of anterior interhemispheric transparaterminal gyrus [11]. These reported approaches able to access tumor successfully with no reported motoric or language deficits after the surgery [10,11].

In this case, transcortical approach by the kocher's point was chosen to access the tumor through the lateral ventricle (Fig. 2). Open surgery was opted for this patient due to the presence of elevated intracranial pressure signs on admission. The tumor was able to be identified at the floor of the frontal horn, thus avoiding resection of the normal brain tissue. After adequate tumor sample was retrieved, the tumor was debulked using CUSA. As the goal of the surgery is to take tumor sample, whilst preserving the patient's motor function, the tumor was resected subtotally. Following the surgery the patient had preservation of his motor and language function.

The tumor pathological anatomy resulting in germinomas, thus the patient was referred for radiotherapy. The management recommendation for this basal ganglia germinoma had been diverse. As this tumor is usually occurs in pediatric population, many suggest a chemotherapy only approach, to avoid the devastating consequences of radiotherapy. However, in adult patients, some suggest the use of radiotherapy only approach [12]. The radiotherapy can be either craniospinal irradiation, or whole brain radiotherapy. Foote et al., reported the clinical outcomes of germ cell tumor radiotherapy in adults with median age of 24.1 year. This study reported the use of craniospinal irradiation of 25 Gy with a boost dose to the tumor of total 40 Gy, resulting in no relapses and mortality after a median follow up of 10.9 years [13]. However there has been no prospective clinical trials for this population, as basal ganglia germ cell tumor in adult is rare.

## 4 Conclusion

Basal ganglia germinoma in adult is a rare occurrence, and due to its location, the surgical approach to access the mass should be individualized in each patient. Transcortical approach from the left kocher's point was a safe and accessible approach for our patient. As the tumor was close to important structures such as hypothalamus, thalamus, and basal ganglia, only partial excision was done. As the pathological anatomy resulting in germinoma. The patient was thus referred for radiotherapy and had resolution of his complaints.

## Provenance and peer reviews

Not commissioned, externally peer-reviewed.

## Consent

Written informed consent was obtained from the patient for publication of this case report and accompanying images. A copy of the written consent is available for review by the Editor-in-Chief of this journal on request.

## Ethical approval

Ethical approval for this study was waived by Ethics Committee of Soetomo Academic General hospital because the anonymity of patient was assured and no more than two subjects were included in this study.

## Funding

None.

## Author contribution

• Tedy Apriawan – data collection, revising, and reviewing the final version of the article
• Vega Pangaribuan – manuscript writing, study conception, patient contribution, article revising, figures creation, study oversight
• Muhammad Azzam – study conception, article revising

## Guarantor

Tedy Apriawan accepts full responsibility for this review manuscript.

## Research registration number

Not applicable.

## Conflict of interest statement

The authors report there are no competing interests to declare.
